# Supplementary figures and images for: Adipose triglyceride lipase protects renal cell endocytosis in a Drosophila dietary model of chronic kidney disease
Source: PLoS Biol. 2021 May 4;19(5):e3001230. doi: 10.1371/journal.pbio.3001230 (PMC8121332; doi:10.1371/journal.pbio.3001230)

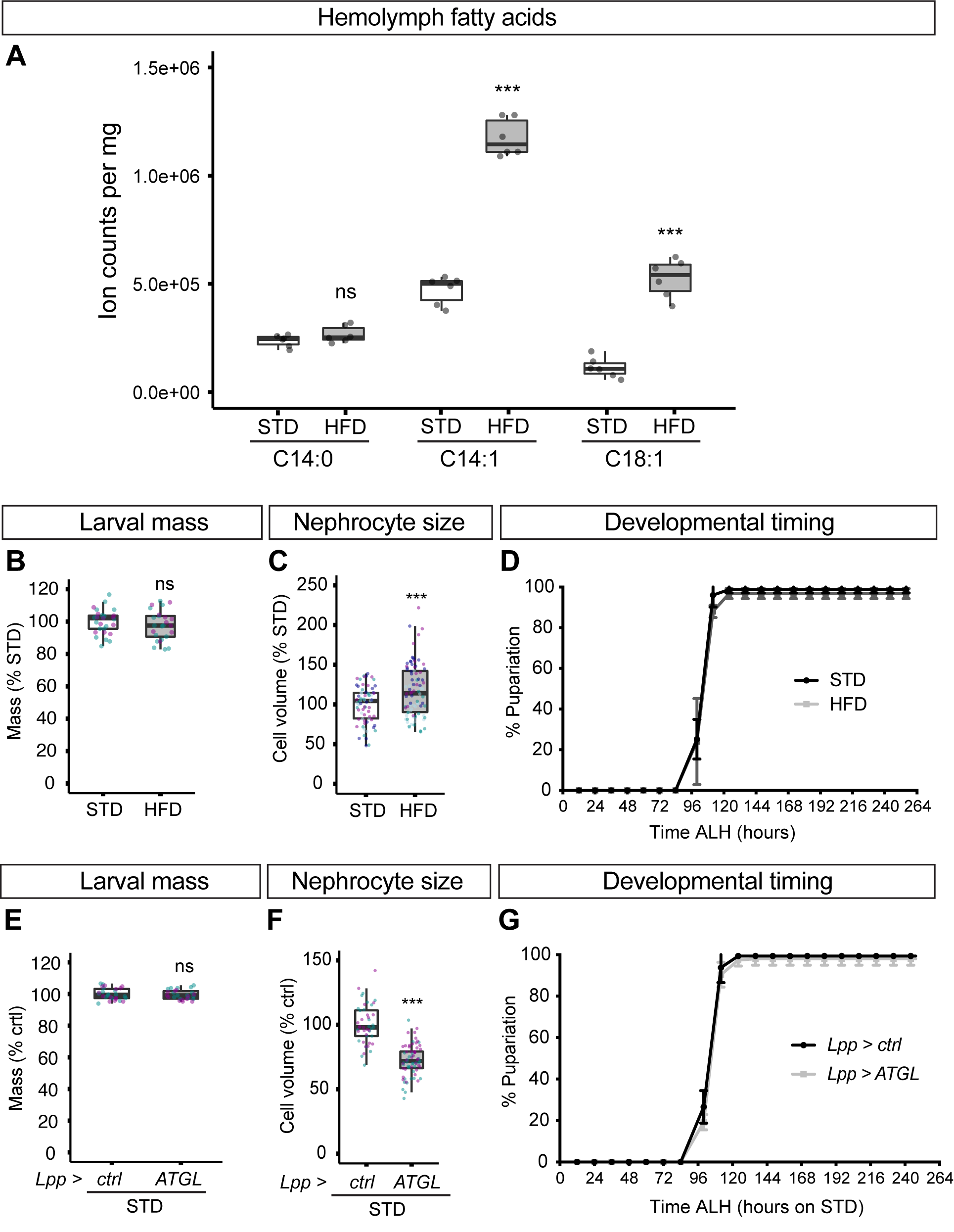

Supplement: S1 Fig — (A) Hemolymph abundances of myristic (C14:0), myristoleic (C14:1), and oleic (C18:1) acids in STD and HFD larvae. Abundance (ion counts per mg) was measured by GC–MS and normalised to larval wet weights. (B–D) Graphs compare STD and HFD animals, indicating that they have similar larval weight (mg), nephrocyte volume (μm3), and developmental timing (% pupariation versus hours after larval hatching). Note that nephrocyte size is significantly different (p < 0.0005) between STD and HFD animals. (E–G) Graphs compare STD animals expressing ATGL in the fat body (Lpp>ATGL) with controls (Lpp-GAL4). indicating that they have similar larval weight (mg), nephrocyte volume (μm3), and developmental timing (% pupariation versus hours after larval hatching). Note that nephrocyte size is significantly different (p < 0.0005) between control and ATGL expressing animals. See S1 Data for details of p-values and the type of statistical model used for all graphs in this study. S2 Data provides the source data used for all graphs and statistical analyses. ATGL, adipose triglyceride lipase; GC–MS, gas chromatography–mass spectrometry; HFD, high-fat diet; STD, standard diet. (TIF) [file pbio.3001230.s001.tif]

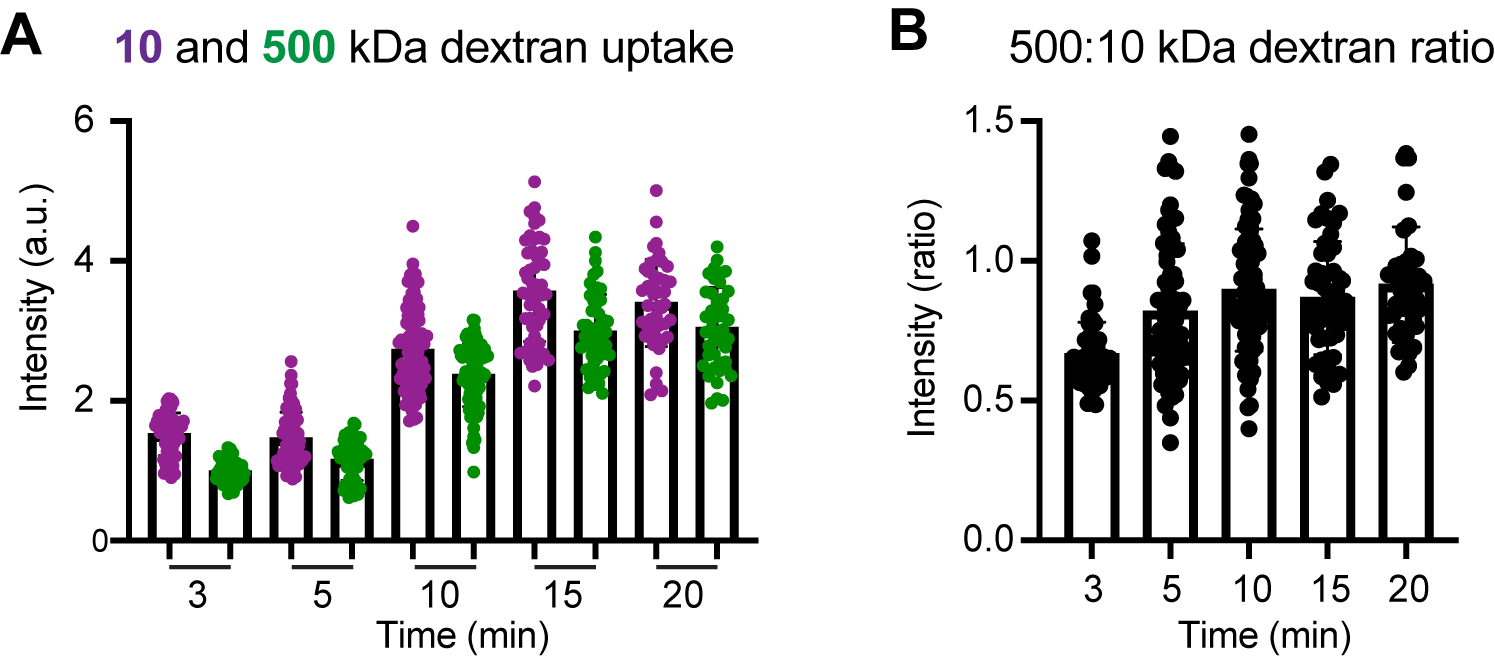

Supplement: S2 Fig — (A, B) Graphs quantify individual uptake (A) and uptake ratio (B) of fluorescently labelled 500-kDa and 10-kDa dextrans as a function of time (min) for ex vivo pericardial nephrocytes. See S1 Data for details of p-values and the type of statistical model used for all graphs in this study. S2 Data provides the source data used for all graphs and statistical analyses. (TIF) [file pbio.3001230.s002.tif]

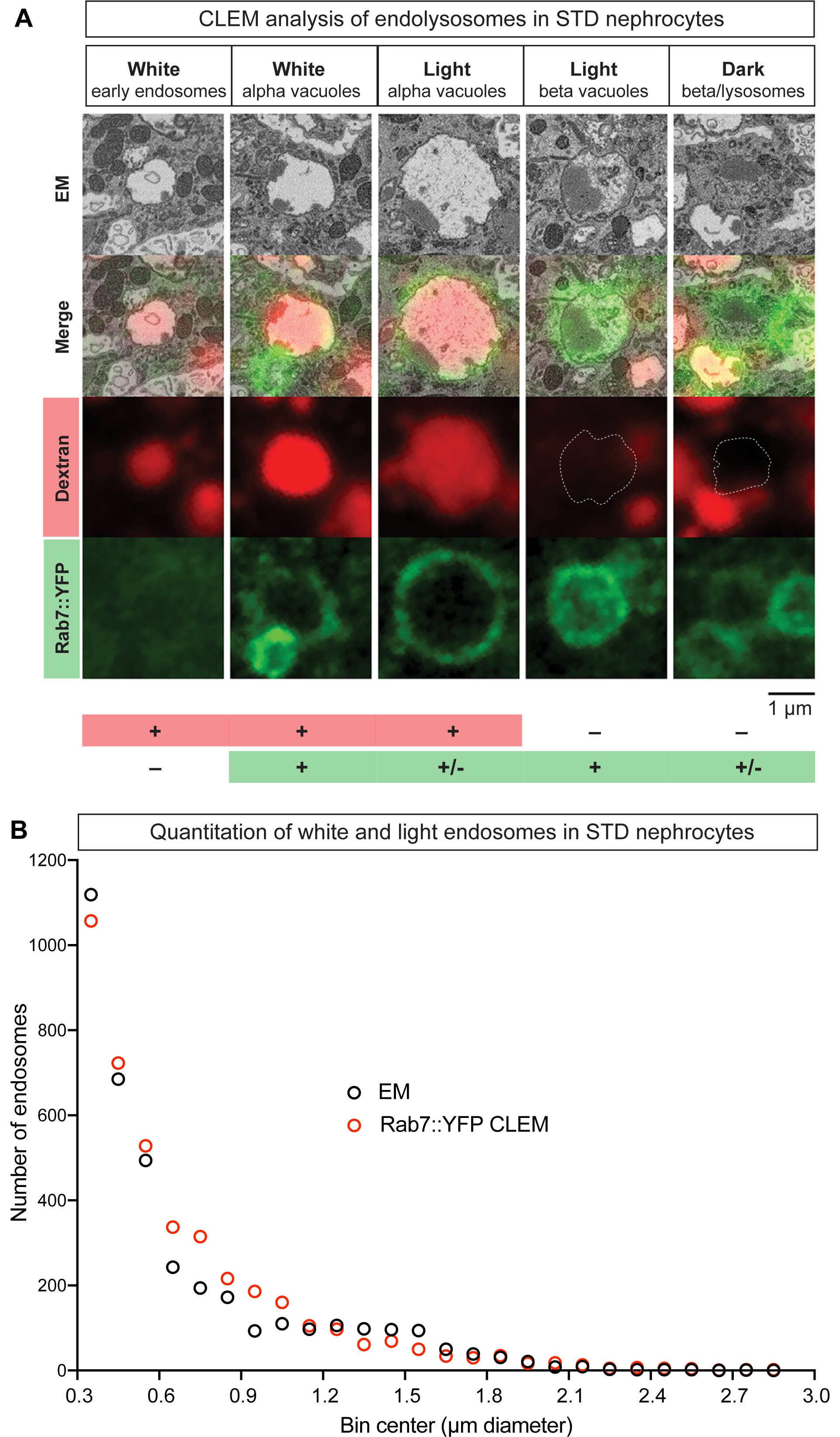

Supplement: S3 Fig — (A) The 5 endolysosomal categories distinguished in CLEM analysis of Rab7::YFPmyc STD nephrocytes subjected to dextran uptake assays. The criteria used were scanning EM luminal density (“white,” “light,” or “dark”), and also the “+” or “−” status of expression of Dextran and Rab7::GFP. Scale bar = 1 μm. (B) Quantitations from SBF SEM volumes of a STD control and a STD Rab7::YFPmyc nephrocyte showing similar endosome size distributions. Note that “white” and “light” endosomes but not “dark” endolysosomes were segmented and quantified per cell according to their diameter (μm). See S1 Data for details of p-values and the type of statistical model used for all graphs in this study. S2 Data provides the source data used for all graphs and statistical analyses. CLEM, correlative light-electron microscopy; SBF SEM, serial blockface scanning electron microscopy; STD, standard diet. (TIF) [file pbio.3001230.s003.tif]

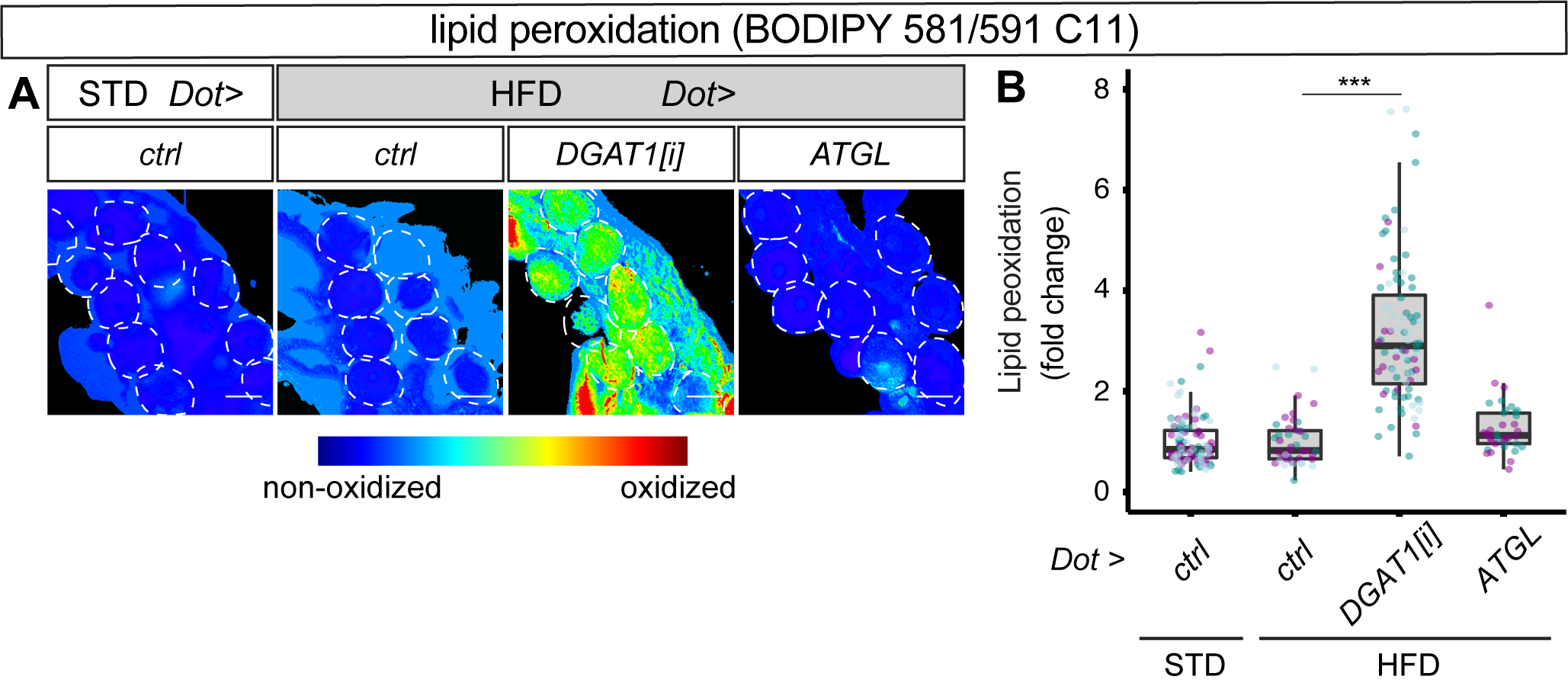

Supplement: S4 Fig — (A) Confocal panels represent ratio of oxidised (500- to 540-nm emission) to non-oxidised (570- to 610-nm emission) forms of the lipid peroxidation sensor BODIPY 581/591 C11 in pericardial nephrocytes (dotted outlines) from STD larvae and HFD larvae carrying Dot>DGAT1[i] or Dot>ATGL. (B) Graph quantifies oxidised:non-oxidised ratios of the lipid peroxidation sensor BODIPY 581/591 C11 in pericardial nephrocytes for the dietary and genetic manipulations in A. On HFD, lipid peroxidation is increased by DGAT1 knockdown but not by ATGL expression. See S1 Data for details of p-values and the type of statistical model used for all graphs in this study. S2 Data provides the source data used for all graphs and statistical analyses. ATGL, adipose triglyceride lipase; DGAT1, diglyceride acyltransferase 1; HFD, high-fat diet; STD, standard diet. (TIF) [file pbio.3001230.s004.tif]

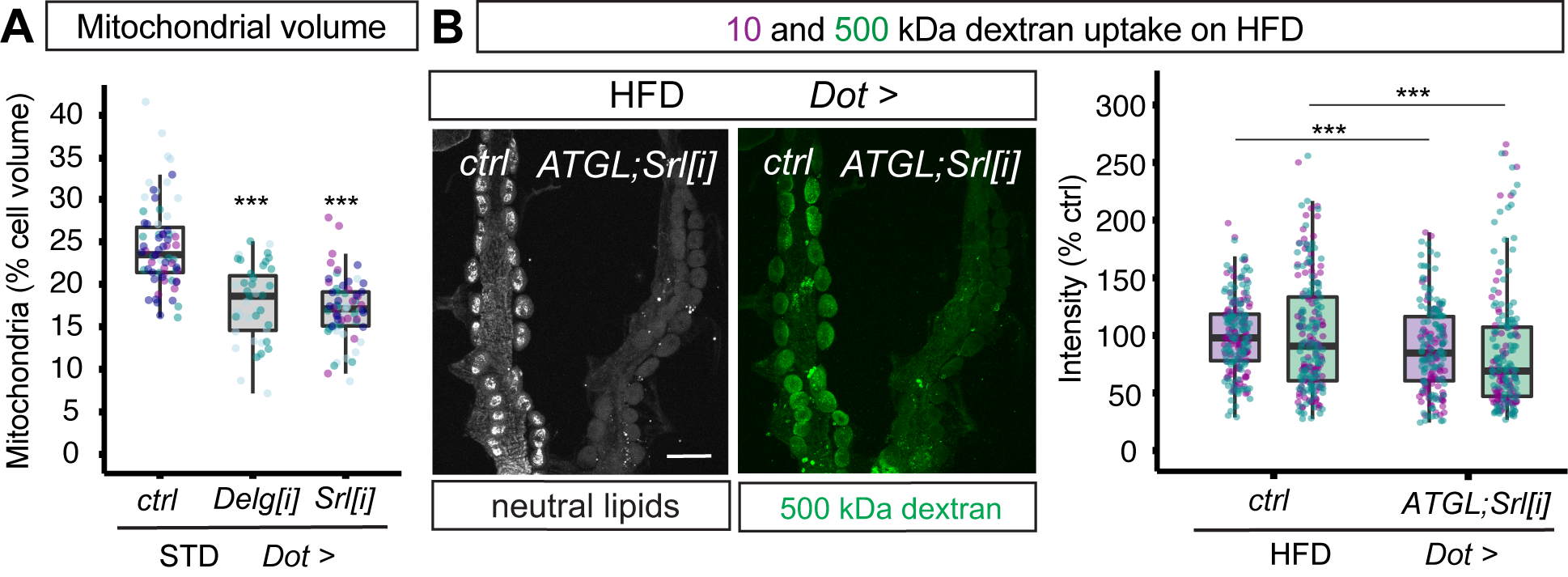

Supplement: S5 Fig — (A) Delg or Srl knockdown decreases mitochondrial volume in STD nephrocytes. Quantitation of mitochondrial volumes (as % of cell volume) of STD pericardial nephrocytes for control (Dot>ctrl), Dot>Delg[i] and Dot> Srl[i] larvae. (B) Confocal panels show 10-kDa and 500-kDa dextran uptake in ex vivo pericardial nephrocytes of control (Dot>ctrl) and Dot>ATGL; Srl[i] larvae on HFD. The dextran signals of both genotypes, imaged within the same field of view, are comparable. Graph shows small but significant decrease in 10-kDa and 500-kDa dextran uptake between control (Dot>ctrl) and Dot>ATGL; Srl[i] larvae on HFD. Note that in the absence of Srl knockdown, ATGL significantly increases dextran uptake on HFD. See S1 Data for details of p-values and the type of statistical model used for all graphs in this study. S2 Data provides the source data used for all graphs and statistical analyses. ATGL, adipose triglyceride lipase; HFD, high-fat diet; Srl, Spargel; STD, standard diet. (TIF) [file pbio.3001230.s005.tif]
