# Supplementary material for: Adipose triglyceride lipase protects renal cell endocytosis in a Drosophila dietary model of chronic kidney disease
Source: PLoS Biol. 2021 May 4;19(5):e3001230. doi: 10.1371/journal.pbio.3001230 (PMC8121332; doi:10.1371/journal.pbio.3001230)
Supplement: Attachment — Submitted filename: Gould-rev-response-reviewers.pdf [file pbio.3001230.s012.pdf]

PBIOLOGY-D-20-02824R1.
Adipose Triglyceride Lipase protects the endocytosis of renal cells on a high fat diet in *Drosophila*

Reviewer 1:
The study by Lubojemska and colleagues starts out with developing a Drosophila nephrocyte-based model for mammalian chronic kidney disease (CKD). Nephrocytes of fly larva on high fat diet accumulate lipid droplets and also recapitulate several other aspects of CKD including ER and mitochondrial deficits as well as compromised endocytosis. ATGL/bmm overexpression in the fat body of larvae on standard diet causes similar phenotypes. Tissue-specific impairment of TAG synthesis or excessive TAG mobilization clears LDs but has very different consequences on nephrocyte function. Only ATGL/bmm overexpression rescues the adverse effects of HFD. In a final set of experiments the authors implicate ATGL/bmm transcriptional control by HFD. Also, they demonstrate a role of mitochondrial regulators PGC1alpha/Spargel and Delg in HFD-induced nephrocyte dysfunction.

The outline of this highly interesting and well-written study is clear, the experiments are technically sound and - with exceptions - provide convincing evidence for the conclusions drawn. The CLEM studies are impressive, and the statistical treatment of the data is excellent. The figures are clear and informative. Yet, the readers` desire to understand the mechanistic connections tying the correlative phenotypes together are not fully satisfied.

As outlined below, the experiments concerning the transcriptional control of ATGL/bmm are not convincing. A further major drawback is the lack of any direct evidence for the hemolymph lipids (FFAs?, DAG?) the authors imply to cause the pathophysiological effects in nephrocytes in larva on HFD or in larva on normal food, which over-express ATGL/bmm in the adipose tissue.

The elegant genetic experiments leave of lot of space for interpretations concerning the underlying lipid-based mechanisms, which cause nephrocyte malfunction on HFD. The possible consequences of DAGT1/mdy knockdown deserve a broader discussion given the central role of DAG as signaling lipid and intermediate of phospholipid metabolism.
We thank the reviewer for their comments that the study is highly interesting, well written, the experiments technically sound, the genetic experiments elegant, the figures informative, and the statistical treatment excellent.

In the responses to the specific criticisms below, we do our best (given covid19 restrictions) to satisfy the desire to better understand the mechanistic connections, including conducting new experiments. As requested, in the Discussion we have included the potential roles of DAGs as signalling lipids and intermediates of phospholipid metabolism.

Comments (in the order of appearance):

1) Comparing the mammalian and fly renal systems in Fig. 1A is informative. But various structures and cell types referred to in the text are missing and need to be added.

It also remains unclear how a discontinuous nephrocyte/Malpighian tubule system works compared to a nephron.
We thank the reviewer for their helpful suggestion and have added some extra elements to Fig. 1A as well as modifying it to show that hemolymph acts as the conduit to "join up" the discontinuous nephrocytes/malpighian tubule system of *Drosophila*. The Reviewer raises the question of how a discontinuous nephrocyte/Malpighian tubule system actually works. It is thought that nephrocytes can deal with unwanted hemolymph components in at least two ways. First nephrocytes convert some waste molecules into metabolites that are secreted back into the hemolymph to be excreted via the malpighian tubules (PMID: 19783135). Second, nephrocytes can store and sequester toxic hemolymph molecules (e.g metals such as silver), which accumulate throughout the relatively short lifespan of flies compared to most mammals (PMID: 18971929 and PMID: 28164240). As the Reviewer comments, however, much remains to be learned about how the insect renal system functions.

2) Authors state that fat-body specific ATGL expression did not substantially alter nephrocyte size (line 207/208). Yet, the provided data (Fig. S1E) indicate a ~25% decrease in cell volume upon ATGL expression. This should be stated in the main text as it remains a formal possibility that decreased endocytic uptake occurs secondary to decreased cell volume.
We thank the Reviewer for pointing out the error on line 207/208, which has now been corrected in the main text.

3) Along these lines: How is nephrocyte cell volume affected by Dot-Gal4-mediated DGAT1 knockdown or ATGL overexpression specifically in nephrocytes?
Nephrocyte size does not noticeably change with DGAT1 knockdown or ATGL overexpression, although small changes cannot be ruled out as we have not precisely quantified those particular cell volumes. Either way, any small changes in nephrocyte volume would not alone account for the main result here - namely a large (90-100%) increase in dextran endocytic uptake for ATGL overexpression on HFD (Fig 5F). We have also clarified in the revised Materials and Methods that the dextran and albumin endocytic uptake assays measure mean fluorescent pixel intensity across a nephrocyte region-of-interest (rather than the total signal per nephrocyte) and are therefore less sensitive to cell volume changes.

4) Fig. 2A: There seems to be no correlation between dextran uptake and lipid loading. Why?
Reviewer 1 is absolutely correct and this also relates to the second point of Reviewer 2. Our renal lipid droplet model (see Discussion) plus a progressive development of renal disease may, at least in part, explain why all HFD nephrocytes have abundant lipid droplets but not all have strongly decreased dextran uptake. In this scenario, nephrocyte lipid droplets would be induced as part of an early response to HFD that is initially able to safeguard endocytosis in all nephrocytes. Despite lipid droplets, however, progressively longer HFD exposures (and/or higher fatty acid concentrations) would lead to damage accumulation (e.g mitochondrial) above a certain threshold in progressively more and more nephrocytes, as these cells are not turned over during development. Unfortunately, to address the Reviewer's why question with mechanistic rigour would require an extensive series of new experiments including different HFD protocols, timecourses, genetic manipulations etc.

5) Fig. 2C: There seems to be no lipid loading in response to HFD. Why?

In fact, there is strong neutral lipid loading (i.e lipid droplets) in response to HFD. The panels are a little small, so to help increase visibility the brightness of both the STD control and HFD diet have now been increased.

6) Fig. 3 refers to Rab7::GFP not YFP.
We thank the Reviewer for pointing out the error and have now corrected it in Fig 3 and S3 Fig.

7) ESSENTIAL FOR REVISION: The authors invoke but never show increased hemolymph free fatty acid (or DAG, as in fact the BODIPY C12 experiments argue against a contribution of free fatty acids) to be involved in the tissue communication between adipose tissue and nephrocytes on HFD or upon adipose tissue ATGL/bmm overexpression on STD. This needs to be experimentally shown.
This is a valid point that has been technically challenging for us to address experimentally, especially with covid-related delays and associated downtime of the GC-MS instruments. Nevertheless, we have been able to develop a GC-MS assay for measuring individual fatty acids (free and in DAG) in larval hemolymph. Using this new assay, we conducted two independent experiments that both showed large and statistically significant increases in hemolymph fatty acids in the larvae raised on a HFD and one of these experiments is shown in revised S1A Fig). We thank the Reviewer for prompting us to do this experiment, which strengthens our original conclusions. We have revised the text and Materials and Methods accordingly.

8) Is there a formal prove that Lpp-Gal4 does not drive in nephrocytes?
We have never detected any *Lpp>GFP* expression in larval nephrocytes. Importantly, even if the fat body driver *Lpp-GAL4* were to be expressed weakly at some point in nephrocytes, this would not account for our *Lpp>ATGL* phenotype as it is opposite to the one with the nephrocyte driver (*dot>ATGL*). In other words, nephrocyte lipid droplets strongly increase with *Lpp>ATGL* but strongly decrease with *dot>ATGL*.

9) What does "Lpp> " and "Dot> " refer to? Driver line only or crossed to (which?) control?
It refers to the Gal4 driver line crossed to a control as specified in the Materials and Methods. In line with the Reviewer's comments, we have made it clearer when the control was w^{1118} iso^{31} or *UAS-mCherry RNAi.* We also refer to our response to the related Point 18 below.

10) Line 208 (S1D-S1F Fig.) needs attention.
We thank both Reviewers for pointing out this error and have now corrected it.

11) The two left panels in Fig. S4 show signal of (non-oxidized) BODIPY 581/591 C11 outside the cells in a nephrocytes preparation i.e. extracellularly. How is this possible?
The non-nephrocyte signal actually corresponds to other cells (including those of the dorsal vessel) that are dissected together with the pericardial nephrocytes in the same "dorsal midline" preparation. This minimal dissection method avoids unnecessary damage to the nephrocytes.

12) Line 301/2: Statement on the HFD-dependent regulation of ATGL/bmm needs support of a reference.

We have now cited Diop et al., who reported HFD-dependent downregulation of ATGL/bmm mRNA at the level of whole adult flies.

13) ESSENTIAL FOR REVISION: Line 304: What is the evidence for a transcriptional regulation? Post-transcriptional regulation or translational control of GFP protein in the physiologically compromised nephrocytes from HFD animals could plausibly account for differences in fluorescence intensity. Fig. 6A-D data are not providing any convincing evidence and need to be complemented by additional experiments addressing transcriptional regulation more directly.

The Reviewer raises a valid point and, in line with the Editors comments, we have revised the Results text to indicate that the *bmm-GFP* results "suggest" rather than "show" *ATGL* gene regulation and we have also included more explicitly in the Discussion the possibility of post-transcriptional regulation:

"*However, additional evidence is required before it can be determined whether the observed ATGL reporter effects are specific for ATGL transcription or reflect more general changes in gene expression at either the transcriptional or post-transcriptional levels.*"

In addition to the textual changes, we provide the Editors and Reviewers with some data from an additional experiment (Figure R, pasted below), addressing the question of whether post-transcriptional regulation of GFP may contribute to the HFD decrease in nephrocyte Bmm-GFP expression. In this experiment, larvae carried both Bmm-GFP and a control histone::RFP line (His2Av::RFP1). Plotting the nephrocyte GFP/RFP ratio indicates that chronic exposure to a HFD decreases the Bmm-GFP reporter significantly more than that of the histone::RFP reporter. However, these results also show that HFD does modestly decrease the absolute expression levels of histone::RFP. The data in Figure R are therefore consistent with our original conclusion that HFD significantly decreases Bmm-GFP reporter expression but, perhaps not surprisingly, do also suggest that this downregulation is wider than just Bmm transcription. Given that this experiment does not distinguish clearly between transcriptional and post-transcriptional effects and because of covid we have only been able to do it once, we would prefer not to include it in the manuscript itself.

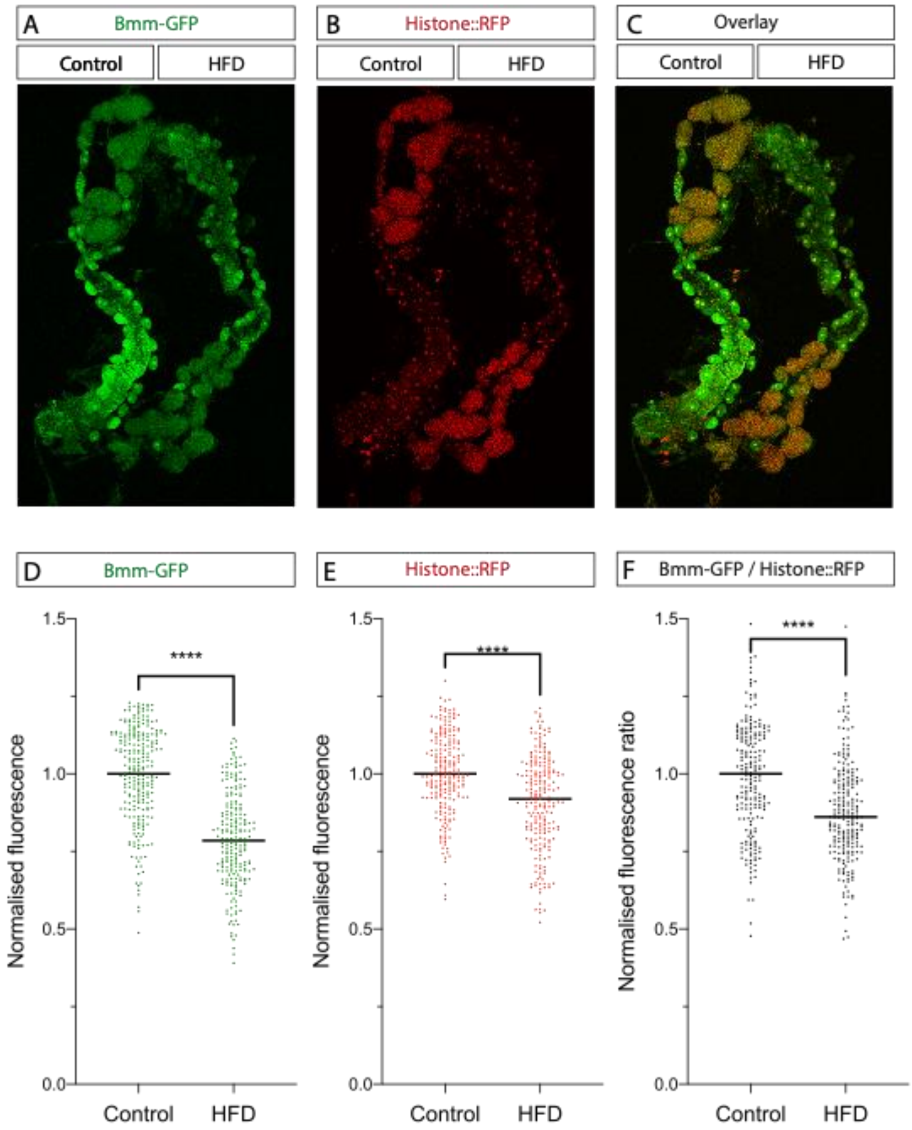

Figure R. HFD represses nephrocyte expression of Bmm-GFP significantly more than Histone::RFP
Representative confocal images of dissected nephrocyte preps for Bmm-GFP (A), Histone::RFP (B) and the merged overlay (C) for control (standard diet) and HFD (high-fat diet) larvae. Mean nuclear fluorescent intensity was determined for each nephrocyte using Volocity, and is plotted for individual nephrocytes pooled from 9 larvae per condition, normalised to control (STD diet): Bmm-GFP (D), Histone::RFP (E) and Bmm-GFP/ Histone::RFP ratio (F). Asterisks indicate $p<0.0001$ in a two-tailed t-test for each control-versus-HFD comparison.

14) Line 316: Introduce GABPA
Thank you for spotting this, we have now introduced GABPA.

15) Fig. 5 (significant difference) and 6E (not difference) data on Dot>ctrl and Dot>DGAT[i] are contradictory. Why? This difference questions the effect of PQQ in DGAT knockdown conditions.
Fig. 5C did overall (three independent experiments) give a significant decrease using a general linear mixed model but, as we stated in the original text, the magnitude of the change was small. We therefore did not make any strong conclusions from this in the original text, especially because, as the Reviewer rightly points out, the comparison between the same genotypes (but different multiple independent experiments) in Fig. 6E was not statistically significant. In line with the Reviewer's comments, we have further clarified the results text and legend referring to Fig 5C. Importantly, these Dot>ctrl and Dot>DGAT1[i] comparisons of HFD mitochondrial volume do not question the effect of PQQ in DGAT1 knockdown because, regardless of whether there is no change or a minor *decrease*, our conclusion that PQQ gives a substantial and significant *increase* to both genotypes will remain entirely valid.

16) Fig. 5A appears to show a fuzzy neutral lipid staining in Dot-ATGL nephrocytes. Why is that?
This weak fuzzy LipidTOX signal is either background staining or it reflects some type of lipid species distributed uniformly in nephrocytes. Either way, the fuzzy signal does not correspond to lipid droplets and so our conclusions are valid.

17) Why does mitochondrial cell volume of Dot-ATGL differ by about 5% between 5C/6E and 6F? This is relevant as it is the unusual high Dot-ATGL value in 6F, which makes Delg[i] and Srl[i] significantly different.
The Reviewer is correct that there are differences between the mean *Dot>ATGL* values for mitochondrial cell volumes in 5C versus 6E versus 6F. These can be accounted for by variations from independent experiment to experiment - which were clear to see, even within a single boxplot. Likely biological sources of this variation include small differences in larval size/stage from experiment to experiment (although these are minimised by synchronization at L1 hatching). There may also be technical sources of variation, from experiment to experiment, that may arise from the confocal microscopy (although, as stated in the Methods, for any one experiment *samples for direct quantitative comparison were imaged on the same day using the same settings*). Importantly, we have accounted for the variance both within and between experiments by using linear mixed models in our statistical analysis and by being transparent in our graphs - marking the individual data points from each experiment in a different colour. Our conclusions about the significant changes in Figure 6F are therefore supported by a robust statistical analysis that takes into account differences from experiment to experiment.

18) Figure 5 (and elsewhere): How can the authors be sure that reversal of the ATGL-overexpression effect upon concomitant DGAT1 or Srl knockdown is not due to a simple GAL4 dilution effect? According to the figure caption, ATGL was not co-expressed with ctrl RNAi, which would have been a more appropriate setup.
We did not explain this in enough detail in the original manuscript but we are very sure that the reversal is not a simple GAL4 dilution effect. This is because the ATGL was indeed coexpressed with a control RNAi (the UAS-mCherry RNAi line cited in the Materials and Methods). We have now clarified what "ctrl" means more explicitly in the relevant figures and their legends.

19) Does Srl[i] and Delg[i] also block LD clearance in Dot>ATGL expressing

nephrocytes of HFD specimens? This would be in line with a reduction of flux through the LD compartment in cell with compromised mitochondrial function in spite of increased lipolytic capacity.
No, it does not block LD clearance. We showed that, on a high fat diet, ATGL; Srl[i] nephrocytes lack lipid droplets, just like ATGL alone (see confocal panels in S5B Fig). This result is compatible with our model (see Discussion) that ATGL-dependent lipolysis acts upstream of the Srl-dependent mitochondrial processes required for optimal nephrocyte endocytosis.

20) The authors are encouraged to add fly stock numbers for those strains received from stock centers.
We have now added the relevant stock numbers.

21) Do nephrocytes accumulate LDs under starvation, when ATGL/bmm gets physiologically up-regulated in the adipose tissue?
This is another interesting question that we do not yet know the answer to.

22) Does DGAT1/mdy overexpression in nephrocytes of HFD larva increase LD size/number and protect against HFD-induced phenotypes mitochondrial and endocytosis phenotypes? This finding would strengthen the argument that lipid flux into the LD compartment acts protective.
We have not tested the effects of DGAT1/mdy overexpression and, as the reviewer says, this could be informative. However, at least for the fat body, it is known that UAS-mdy expression is not sufficient to increase triglyceride accumulation (PMID: 33227003), suggesting that overexpression of the DGAT1 mRNA may not necessarily yield more DGAT1 enzyme activity.

23) Comment on the SI: the supplementary information is informative and complements the main text/figures
Many thanks.

Reviewer 2:

In this paper the authors demonstrate a high fat diet (HFD) induces lipid droplet formation/accumulation in nephrocytes. They go on to show the HFD results in nephrocyte phenotypes including a substantial reduction in ER and mitochondrial volumes. These data reveal strong phenotypic similarities between the nephrocyte model and podocyte/proximal tubule in animals/patients on HFDs. These data suggest the nephrocyte is a powerful model to dissect the role of lipids in pathogenesis of CKD.

Nephrocytes are prodigious endocytosers. The authors go on to show these endocytic functions are highly attenuated by a HFD by assaying for endocytosed material and by quantifying endosome number. Further, evidence is provided that lipid droplets might be caused from overflow from fat body and subsequent take-up by nephrocytes. Take-up in nephrocytes is shown to be Cubulin-dependent.

The authors go on to elegantly dissect the question of the contribution of lipid droplets on HFD-induced renal dysfunction using a combination of cell-type specific knock-down/activation and epistatic studies. They show that ATGL expression but not DEGAT1 knock-down is sufficient to rescue HFD-induced phenotypes in nephrocytes, and go on to define the pathway of the ATGL rescue.

The data is comprehensive, strong and supports the interpretations made. Overall, I thought the approach was very elegant and the manuscript very well argued. I would strongly recommend publication in your journal. I don't suggest further experiments.

Given the high prevalence of CKD and a lack of understanding into the contribution of lipotoxicity in the disease it is likely to be of wide interest to readers of PLoS. The authors describe a new model to study HFD-induced CKD and use cell-type specific genetic manipulations, epitasis experiments and pharmacology to illustrate how powerful this model can be in revealing disease pathways/mechanisms.

We thank this reviewer for their very positive comments on the manuscript and for pointing out the power of the model.

Two minor points the authors might choose to consider:
(1) The authors comment briefly on changes to nephrocyte size: an increase in response to HFD (line 88, S1B); and a decrease in Lpp>ATGL animals (line 208, S1E). Both changes are significant and might influence quantification of parameters measured in the experiments. Did the authors account for this? Perhaps an absolute count of lipid droplet number would be useful to include alongside (if cell size is scaling independently in these experiments).

We did consider this interesting point during the early stages of the project but the results indicated that those two alterations in nephrocyte size alone cannot explain the observed changes in the parameters we measured. For example, lipid droplets are quantified as the percentage of total nephrocyte volume that they occupy, so this measurement should be fairly robust to changes in absolute cell size. In addition, on HFD, there is roughly a 700% increase in lipid droplets whereas cell volume increases by less than 20%. For Lpp>ATGL, a similarly huge increase in lipid droplets is observed whereas cell volume only shrinks by roughly 30%. Nevertheless, the Reviewer's point does raise an important question for the future, which is whether the machinery regulating cell size (e.g the TOR pathway) may also influence

nephrocyte parameters such as lipid droplets or endocytosis.

(2) Might it be interesting to consider correlation between lipid droplets and phenotypes (for e.g. mitochondrial and ER organelle reduction) on a cell-by-cell basis, i.e. do more droplets mean stronger phenotype?

We agree with the reviewer that this kind of detailed "paired" analysis is a potentially interesting point to consider. Based on our available data, however, there does not seem to be a very obvious nephrocyte-by-nephrocyte correlation between lipid droplet abundance and the strength of the dextran endocytosis phenotype that we used as a readout for nephrocyte function (e.g see HFD panels in Fig2A). We therefore decided not to look into nephrocyte-by-nephrocyte correlations for lipid droplets with mitochondrial/ER volumes.
