# Supplementary material for: Adipose triglyceride lipase protects renal cell endocytosis in a Drosophila dietary model of chronic kidney disease
Source: PLoS Biol. 2021 May 4;19(5):e3001230. doi: 10.1371/journal.pbio.3001230 (PMC8121332; doi:10.1371/journal.pbio.3001230)
Supplement: Attachment — Submitted filename: Gould-rev2-response.docx [file pbio.3001230.s013.docx]

**PBIOLOGY-D-20-02824R2.**

Adipose Triglyceride Lipase protects renal cell endocytosis in a *Drosophila* dietary model of chronic kidney disease

**Editorial Comments**

**Dear Alex, I’ve just realised I forgot to include a suggestion to improve the title, which is currently a bit ambiguous. Please consider the following suggestion: “Adipose Triglyceride Lipase protects renal cell endocytosis in a Drosophila high-fat-diet model of mammalian chronic kidney disease” Many thanks! Ines**

Thanks for the suggestion, we have modified the title in line with your comments to make it less ambiguous with respect to the fact that we are using a fly model of chronic kidney disease.

**Note that we do not require all raw data. Rather, we ask that all individual quantitative observations that underlie the data summarized in the figures and results of your paper be made available in one of the following forms:**

**1) Supplementary files (e.g., excel). Please ensure that all data files are uploaded as 'Supporting Information' and are invariably referred to (in the manuscript, figure legends, and the Description field when uploading your files) using the following format verbatim: S1 Data, S2 Data, etc. Multiple panels of a single or even several figures can be included as multiple sheets in one excel file that is saved using exactly the following convention: S1_Data.xlsx (using an underscore).**

We have now uploaded two Supporting Information data files as S1_Data.xlsx and S2_Data.xlsx. The latter contains multiple sheets in one file.

**2) Deposition in a publicly available repository. Please also provide the accession code or a reviewer link so that we may view your data before publication.**

All the individual quantitative observations are now contained in a new Supporting Information data file S2_Data.xlsx. **Regardless of the method selected, please ensure that you provide the individual numerical values that underlie the summary data displayed in the following figure panels as they are essential for readers to assess your analysis and to reproduce it:

Fig. 1C, E; Fig. 2B, D; Fig. 3B; Fig.4B, D, F, H; Fig. 5A-H; Fig. 6B-G; Fig. S1A-G; Fig. S2A, B; Fig. S3B; Fig. S4B and Fig. 5A, B

NOTE: the numerical data provided should include all replicates AND the way in which the plotted mean and errors were derived (it should not present only the mean/average values).**

All individual numerical values corresponding to the raw data are now provided in an additional spreadsheet, one figure per worksheet (S2_Data.xlsx).

The original file, S1_Data.xlsx, contains details of the statistical models and analysis used for every figure. **Please also ensure that figure legends in your manuscript include information on WHERE THE UNDERLYING DATA CAN BE FOUND, and ensure your supplemental data file/s has a legend.**

We have added the following statement to the legend of Figure 1:

*See* ***S1 Data*** *for details of p values and the type of LMM used for all graphs in this study.* ***S2 Data*** *provides all of the source data used for the graphs and statistical analyses in this study.”*

We have also added this statement to the end of the Materials and Methods:

*Statistical methods, parameters and results for each figure are summarized in* ***S1 Data*** *and the corresponding source data are provided in* ***S2 Data****.*

**Please also provide a blurb which (if accepted) will be included in our weekly and monthly Electronic Table of Contents**

We have now uploaded a blurb.

**Reviewer's Comments
Rev. 1: All the relevant points of concern were satisfayingly address. I congratulate the authors to a very intersting study.**

We thank this reviewer for their kind comments.

**Authors own Corrections**

In the Discussion (line 422), we deleted the citation to ref 55 as this was mis-cited (but the reference list remain unchanged in the bibliography)

In the Discussion (lines 426-435), we also made a few changes to correct a couple of clumsy sentences (see Gould-rev2-manu-track-changes.docx).

**Additional editorial corrections requested in subsequent email of 7/04/21**

**1. Please ensure that the CRediT author contributions listed under each co-author are completed in full. To qualify for authorship, each author must meet at least one of the seven core contributions (conceptualization; data curation; formal analysis; investigation; methodology; software; validation), as well as at least one of the writing contributions (original draft preparation; review and editing). Authors may also be noted against any of the remaining contributions, however these alone will not qualify a contributor for authorship.
-At this stage, the following Authors require contributions: Sebastian Sorge, Andrew P Bailey. Please ensure that the full contributions of each author are acknowledged in the "Add/Edit/Remove Authors" section of our submission form. If they do not meet criteria for authorship please let us know as soon as possible by emailing** [**plosmedicine@plos.org**](mailto:plosmedicine@plos.org)**.**

We have now uploaded the author contributions for the two new authors, Sorge and Bailey. **2. Please provide the editors with a copy of your manuscript which tracks the changes that you have made in order to accommodate the reviewer and editorial comments. This will enable a swift editorial assessment of your manuscript. If you are submitting a PDF please ensure that the changes are clearly highlighted. Please list this file as file type ‘Revised article with changes highlighted’.**

This has now been uploaded as the file Gould-rev2-manu-track-changes.docx **3. Please upload your point-by-point response to all reviewer comments as a separate file from your cover letter. Please upload this to your submission as a "Response to Reviewer" file type.**

This has now been uploaded separately as Gould-rev2-response.docx
